# Clinical efficacy and tolerability of Gabapentinoids with current prescription patterns in patients with Neuropathic pain

**DOI:** 10.12669/pjms.35.6.652

**Published:** 2019

**Authors:** Abida Shaheen, Syed Mahboob Alam, Arsalan Ahmad, Moosa Khan

**Affiliations:** 1 Abida Shaheen, MPhil. Associate Professor, Pharmacology, Shifa College of Medicine, Shifa Tameer-e-Millat University, Islamabad, Pakistan; 2 Syed Mahboob Alam, PhD. Associate Professor, Pharmacology, Basic Medical Sciences Institute, JPMC, Karachi, Pakistan; 3 Arsalan Ahmad, MD. Professor, Neurology, Shifa College of Medicine, Islamabad, Pakistan; 4 Moosa Khan, PhD. Associate Professor, Pharmacology, Shaheed Zulfiqar Ali Bhutto Medical University, Islamabad, Pakistan

**Keywords:** Gabapentinoids, Neuropathic pain, Current dosages, Pain relief, Adverse effects

## Abstract

**Objective:**

To investigate the current dosing regimens of gabapentinoids in Pakistani patients with neuropathic pain and to compare their clinical efficacy and tolerability in terms of pain relief and adverse effects using difference in pain score as a treatment outcome.

**Methods:**

This observational, prospective study was conducted in 320 patients with neuropathic pain from August 2016 to March 2018 at Basic Medical Sciences Institute (BMSI), Karachi in collaboration with Shifa International Hospital and Benazir Bhutto Hospital, Islamabad. Demographic data, treatment-related adverse effects and pain intensity was documented at recruitment and follow up visits at two, four and eight weeks. Discontinuation due to adverse effects and lack of efficacy were also recorded. Data was entered and analyzed using SPSS version 22.

**Results:**

Mean age of patients was 52.57±12.47 and the most common ethnicity were Punjabi speaking population (66%). Diabetic neuropathy (51%) was the most common etiology followed by radicular pain (25%). Mean dosages of pregabalin and gabapentin were 114mg and 470mg respectively. Mean pain score was significantly reduced by gabapentinoids (<0.001). Dizziness, drowsiness and somnolence were frequent adverse effects. Common dosages for pregabalin and gabapentin were 75 mg/day and 300 mg/day respectively.

**Conclusion:**

Current dosing regimens of gabapentinoids in Pakistani patients with neuropathic pain were found to be efficacious at low dosages in comparison to international recommended dosages. Gabapentin and pregabalin were both similar in terms of reducing pain score but onset of pain relief was relatively faster with pregabalin. Dizziness, drowsiness and somnolence were frequently reported with both gabapentinoids; however, visual blurring, ataxia and weight gain were observed only with the use of pregabalin. Adverse effects are frequently observed with gabapentinoids which necessitates reverting back to low dosages or switching to other drugs for pain relief.

## INTRODUCTION

Neuropathic pain (NeP) is defined as pain caused by a lesion or disease of the somatosensory system.^1^,^2^ Multiple heterogeneous etiologies of central or peripheral origin precipitate neuropathic pain and severely affect the quality of life.^3^ Global prevalence of neuropathic pain ranges from 6.9-10% with spinal cord injury accounting for 40% cases.^4^,^5^ The second most common etiology for NeP is diabetic peripheral neuropathy (DPN) which is present in 22-28% of diabetic population.^6^-^9^

The first line treatment options for the management of NeP include gabapentinoids, tricyclic antidepressants, serotonin-noradrenaline reuptake inhibitors and opioids. Pregabalin and gabapentin are the two gabapentinoids which have been approved and widely prescribed for NeP. These are derivatives of the inhibitory neurotransmitter gamma-aminobutyric and bind to presynaptic α_2-_δsubunit of voltage-dependent calcium channels which leads to reduced neurotransmitter release resulting in attenuation of post-synaptic excitability.^10^

Pregabalin has been approved for the treatment of neuropathic pain syndromes with considerable efficacy in dosages range from 150mg up to 600 mg/day depending on patients’ therapeutic response and tolerability. On the other hand, gabapentin is prescribed with starting dose of 300mg and it is then titrated up to 1800-3600mg/day divided in three doses in patients with normal renal function.^11^,^12^ Pregabalin requires less frequent daily dosing and comparable efficacy to gabapentin owing to its higher potency, linear pharmacokinetics and greater bioavailability.^11^,^13^

Most of the clinical studies involving administration of gabapentinoids have reported dizziness, drowsiness and somnolence as the most frequent dose-dependent adverse effects which occur in about every fourth patient. Other common adverse effects include nervousness, headache, blurred vision, dry mouth, peripheral edema, weight gain, constipation, blurred vision, decreased motor coordination and ataxia in 1-10% of patients.^11^,^14^,^15^

Multiple factors make NeP difficult to manage including heterogeneous diagnostic criteria, inadequate response to existing treatment options and variable efficacy of the available drugs. Current clinical practices are based on randomized clinical trials and clear recommendations on dosages are not available.^11^ There are considerable interindividual variations in therapeutic response to gabapentinoids hence prescribing strengths of these drugs also vary and physicians mostly rely on their clinical experience for optimal dosages.^10^,^12^,^14^ Current treatment dosages of gabapentinoids in Pakistani population are different from international guidelines.^12^ Scarce data is available from Pakistan about the clinical efficacy and tolerability of gabapentinoids despite routine clinical use thus observational, noninterventional studies are required to observe the dosage patterns and therapeutic effectiveness of gabapentinoids in clinical practice.

With this background, aim of this study was to explore the current dosing regimens of pregabalin and gabapentin in patients with neuropathic pain. Furthermore, our study also compared the efficacy and tolerability of gabapentinoids in terms of pain relief and adverse effects using difference in pain score as a treatment outcome.

## METHODS

This observational, prospective study was carried out in outpatient pain clinics over a period of 20 months from August 2016 to March 2018 at Shifa International Hospital, Islamabad, Benazir Bhutto Hospital, Rawalpindi in collaboration with Basic Medical Sciences Institute, Karachi. Study protocol was approved by Institutional Review Board & Ethics Committee. Three hundred and twenty patients of either gender, aged ≥ 18 years were included after obtaining written and verbal informed consent with established diagnosis of neuropathic pain due to heterogeneous etiologies. Patients’ pain scores at the time of recruitment were ≥ 40mm on the 100mm Visual Analog Scale (VAS) of the Short Form-McGill Pain Questionnaire and an average pain score ≥4 on an 11-point Numeric Rating Scale. Patients with creatinine clearance less than 60ml/min or any other renal insufficiency and gastrointestinal diseases that could interfere with the absorption of drugs were excluded from the study. Patients’ demographic data, clinical history and all relevant information related to neuropathic pain were recorded in a detailed structured questionnaire.

The patients who were receiving different dosages of pregabalin or gabapentin in QD, BID and TID regimen by prescribing clinicians were placed into dosing groups of pregabalin (50-75, >75-150, >150-200 and >200-300mg) and gabapentin (0-200, >200-300, >300-600, >600-900mg). The patients were followed for primary efficacy outcomes by measuring pain scores by VAS and NRS at baseline and later at 2-, 4- and 8 weeks’ follow-up visits. All the clinical progress and adverse effects since the commencement of these medications were recorded for the full duration of study. Discontinuation of gabapentinoids due to lack of efficacy, inadequate response and adverse effects were also recorded. For this study we estimated the sample size using a previously published paper^9^ which compared the pain scores of gabapentinoids using Visual Analogue Scores. To detect of a difference of 2.28 points in pain scores between pregabalin treated group (previous reported estimate 38.90±5.70) and gabapentin treated group (previous reported estimate: 41.62±4.79); with 95% confidence level and 80% power of the test, we used OpenEpi software and the minimum sample required for this study was 142 with 71 individuals in each group.

### Statistical analysis

Exposure to gabapentinoids and outcomes (clinical efficacy, adverse effects) were recorded for all those patients who continued the treatment till 8-weeks of the study. Clinical efficacy was defined as a change in mean pain score from baseline to a clinically meaningful reduction of ≥30% or ≥50%.^16^ Data was converted to electronic files using the Microsoft Excel Software and Excel files were imported to Statistical Package for Social Sciences (SPSS) version 22 for analysis. The quantitative variables like age, dosage of drugs, time to onset of pain relief and difference in pain scores were presented with Mean and Standard Deviations (SD). The qualitative variables such as ethnicity, gender and adverse effects were reported in frequency and percentage. For making comparison of pain scores, time to relief in pain between groups i.e. pregabalin and gabapentin, we used two independent samples Student ‘t’ test. Statistical significance was set at *p-*value less than 0.05.

## RESULTS

Three hundred and twenty patients were enrolled in the study out of which sixty-eight patients could not complete the study due to various reasons. Sixteen of these patients were lost to follow up during the study whereas twenty-four patients showed inadequate response to gabapentinoids and were switched to other drugs. Twenty-eight patients developed severe dizziness or drowsiness and could not tolerate these adverse effects. Two hundred and fifty-two patients completed the study and were evaluated for efficacy and tolerability measures.

Mean age of these study participants was 52.57±12.47. Most common ethnic group among our study participants were Punjabi speaking population (66%) followed by Pathan (28%) and Urdu speaking participants (6%). Number of females was almost twice as the number of male participants. Results of the demographic data are summarized in Table-I.

**Table I:** Demographic data.

Age (years) mean (SD)	52.57±12.47
***Ethnicity, n (%)***	
Punjabi	1676)
Pathan	7028)
Urdu Speaking	15 (6)
***Gender, n (%)***	
Male	92 (36)
Female	160 (64)

Two hundred and six study participants (81.74%) received pregabalin whereas forty six (18%) patients received gabapentin for different types of neuropathic pain. The most common etiologies for neuropathic pain were diabetic neuropathy and radicular pain with prevalence of 51% and 24% respectively. Frequencies of etiologies, mean dosages and dosage arms of both gabapentinoids are summarized in Table-II.

**Table II:** Dosage groups and mean dosages of Gabapentinoids for different etiologies of NeP.

Etiology	Frequency n (%)	Pregabalin Dose mg/d (n)				Mean Pregabalin dose (mg/d) Mean (±SD)	Gabapentin Dose mg/d (n)				Mean Gabapentin dose (mg/d) Mean (±SD)

		50-75	>75-150	>150-200	>200-300		0-200	>200-300	>300-600	>600-900	
Diabetic Neuropathy	128 (50.7)	81	28	5	1	102.04±46.14	1	7	5	0	363.64±167.87
Radicular Pain	62 (24.60)	17	10	8	2	139.19±70.60	6	4	7	8	578.26±295.15
Intercostal Neuralgia	7 (2.77)	2	2	2	0	154.17±51.03	1	0	0	0	200
CRPS	7 (2.77)	4	1	1	1	128.57±95.12	0	0	0	0	-
Fibromyalgia	6 (2.38)	3	2	0	0	105.00±44.72	1	0	0	0	200
Post stroke neuralgia	2 (0.79)	0	2	0	0	150	0	0	0	0	-
Trigeminal Neuralgia	5 (1.98)	5	0	0	0	85.00±13.69	0	0	0	0	-
Carpal Tunnel Syndrome	5 (1.98)	2	1	1	1	170.00±102.16	0	0	0	0	250±70.71
Guillain Barre Syndrome	3 (1.19)	0	1	0	0	150	1	0	0	1	500±565.69
CKD	9 (3.57)	5	0	1	1	117.86±99.70	1	1	0	0	250±70.71
Post-surgical pain	9 (3.57)	5	1	2	0	115.63±74.33	0	0	0	1	900
Post Chemotherapy	5 (3.57)	1	3	0	0	125.00±50.00	0	1	0	0	300
Others	4 (1.58)	4	0	0	0	81.25±23.94	0	0	0	0	-

Mean dosages of pregabalin in all patients was114.25±59.58 mg whereas mean dosages required by Urdu speaking, Punjabi and Pathan for all etiologies were 101.79±71.03, 114.24±59.58 and 116.56±54.72mg respectively with a p value of 0.293. Furthermore, mean dosage requirements of gabapentin were 470.48±277.45mg in all patients. Mean dosages of gabapentin were 486.96±268.83mg in Punjabi speaking and 555.56±292.02mg in Pushto speaking population with a p value of <0.001.

Dizziness, drowsiness and somnolence were the most frequent adverse effects reported with both pregabalin and gabapentin. Frequencies of adverse effects reported are summarized in Table-III.

**Table III:** Adverse effects and inadequate response reported with Gabapentinoids.

Adverse effects observed in patients who completed the study (n=252)			

Drugs	Adverse effects	n (%)	Mean dose ±SD (mg/d)
Pregabalin	Dizziness, Drowsiness, Somnolence	58 (23)	95.43±53.06
	Visual Blurring	3 (1.19)	125.00±90.14
	Ataxia	4 (1.58)	56.25±10.83
	Weight Gain	1 (0.39)	300
Gabapentin	Dizziness, Drowsiness, Somnolence	10 (3.96)	360±171.27

*Patients’ non-compliance due to severe adverse effects (n=28)*			

	*n (%)*		*Mean dose ±SD (mg/d)*
Pregabalin	15 (54)		112.00±48.17
Gabapentin	13 (46)		375.68±189.70

*Patients with inadequate response (n=24)*			

Pregabalin	7 (29)		126.21±49.20
Gabapentin	17 (71)		325.58±162.73

*Lost to follow up (n=16)*			

Pregabalin	4 (25)		106.28±80.24
Gabapentin	12 (75)		384.38±177.64

The mean pain score for all etiologies using the NRS scale after approximately two weeks’ duration was reduced to 1.92 in patients taking pregabalin and 1.93 in patients receiving gabapentin. These results are summarized in Table-IV.

**Table IV:** Difference in pain score after use of Gabapentinoids.

Drugs	Time to onset of pain relief (days)	p-value	Pain Score Before	Pain score After	Difference	p-value	Overall p-value
Pregabalin	13.73± 1.45	<0.001	5.94±0.90	1.92±0.83	4.02±1.02	<0.001	<0.001
Gabapentin	18.28 ± 2.68		6.17±0.93	1.93±0.68	4.23±1.06	<0.001	

## DISCUSSION

In our study, majority of the participants belonged to Punjabi and Pushto speaking ethnic groups as these groups represent majority of population of Rawalpindi and Islamabad where this study was conducted. Number of female patients was almost twice than males and consistent with other studies conducted to compare prevalence of chronic pain in both genders concluding females have greater pain sensitivity, report pain more frequently and have a lower threshold for most types of pain including neuropathic pain.^17^

The mean dosages of pregabalin and gabapentin for all etiologies were 114.2±59.6 and 470.5±277.45 respectively. Furthermore, pregabalin frequency of administration was more which could be due to discrete pharmacokinetic advantages of pregabalin over gabapentin.^10^,^13^ According to international guidelines, gabapentinoids are minimally effective or ineffective at low dosages.^18^,^19^ In our study, most common dosage of pregabalin was 75mg/day and these findings are in contrast to the international guidelines which suggest minimum starting dose of 150mg and clinically effective dose at 300-600mg/day.^13^,^18^ Similarly, most patients taking gabapentin were given dosages of less than 900mg/day which are in disagreement to the international guidelines recommending 1800-3600mg for effective pain relief.^10^,^11^,^19^ Consistent with our findings, a recent study from Japan using hospital prescription database reported pregabalin daily maintenance dose of 127.8mg which was significantly lower than those reported in the USA and Europe, highlighting different dosage requirements in different populations.^20^

Our results showed that patients taking gabapentin and pregabalin for neuropathic pain showed a significant decrease in pain from a mean pain score of 6.2 to 1.9 and 5.9 to 1.9 after a mean duration of 18.28 and 13.73 days, respectively. These results of our study are in accordance with different studies where both drugs resulted in a mean decrease of pain intensity of approximately 30-50% in majority of the patients.^13^,^21^

Pregabalin was most commonly prescribed for diabetic neuropathy and the results of our study are consistent with other researches proving pregabalin as better choice as monotherapy in 75 to 300 mg/day in early reduction of pain.^13^,^18^ Gabapentin was preferred for radicular pain management. There is limited literature on direct comparison between the two drugs for radicular pain but the results of our study are in accordance with other studies suggesting that gabapentin is more efficacious than pregabalin for radiculopathies and treatment should be commenced with gabapentin though its average daily dose was significantly lower in our study.^22^,^23^

Neuropsychiatric adverse effects such as severe dizziness, drowsiness and somnolence were reported in 23% and 4% of patients receiving pregabalin and gabapentin, respectively. In our study, mean doses of patients with adverse effects were significantly less compared to the average doses in patients who did not report such adverse effects. Moreover, sixty-eight patients were not included for final analysis and main reasons for drop out were non-compliance due to severe adverse effects. Mean dosages of pregabalin and gabapentin were not similar in different ethnic population though not statistically significant. Some studies have suggested role of distinct genetic polymorphism for regulation of proteins for absorption, metabolism, and excretion and as transporter molecule at the site of action in different population which might result in variation of clinical response to these drugs.^24^,^25^

To our knowledge, there have been no studies from Pakistan to evaluate efficacy and tolerability of gabapentinoids in chronic NeP. We acknowledge potential limitations of the study as it was observational, time-bound and conducted in running OPDs hence it was not possible to categorize patients in equal groups for head to head comparison as the drugs and doses were selected by clinicians according to type and severity of NeP. However, potential strengths of our study were adjustment of other variables such as detailed documentation, regular follow-up and close monitoring of adverse effects.

## CONCLUSION

In summary, effective dosing range of pregabalin and gabapentin was 114.2±59.6 ad 470.5±277.45mg/d, respectively. Mean time in onset of relief of pain was less for pregabalin as compared to gabapentin (<0.001). Pregabalin prescription rate was higher as compared to gabapentin. Majority of patients experienced dizziness, drowsiness and somnolence at low doses. Our study findings conclude that Pakistani patients respond to neuropathic pain at low dosages in comparison to international recommendations. Given routine clinical use of gabapentinoids, future clinical trials are required to define appropriate dosing regimens and molecular studies are needed to explore the role of polymorphism in Pakistani population and occurrence of adverse effects at low dosages.

### Authors’ Contribution:

**AS** conceived, designed, collected data, did statistical analysis and drafted manuscript.

**SMA and MK** drafted manuscript, reviewed and did final approval of manuscript.

**AA** conceived and designed study, collected data and reviewed manuscript.
